# miR-423-5p and miR-92a-3p in Alzheimer’s disease: relationship with pathology and cognition

**DOI:** 10.3389/fnagi.2025.1637368

**Published:** 2025-07-22

**Authors:** Sang-Won Han, Young Ho Park, Jung-Min Pyun, Paula J. Bice, SangYun Kim, Andrew J. Saykin, Kwangsik Nho

**Affiliations:** ^1^Department of Neurology, Soonchunhyang University Seoul Hospital and Soonchunhyang University College of Medicine, Seoul, Republic of Korea; ^2^Department of Neurology, Seoul National University Bundang Hospital and Seoul National University College of Medicine, Seongnam-si, Republic of Korea; ^3^Department of Radiology and Imaging Sciences, Center for Computational Biology and Bioinformatics, Indiana Alzheimer’s Disease Research Center, Indiana University School of Medicine, Indianapolis, IN, United States

**Keywords:** Alzheimer’s disease, cognitive decline, machine learning, MicroRNAs, miR-423-5p, miR-92a-3p

## Abstract

**Background:**

MicroRNAs (miRNAs), small and highly conserved non-coding RNA molecules, have emerged as promising molecular biomarkers due to their regulatory roles in gene expression and stability in blood.

**Methods:**

We used measurements of 64 plasma miRNAs from 145 participants in the Alzheimer’s disease Neuroimaging Initiative cohort, including 74 probable AD patients and 71 cognitively normal (CN) older adults. We performed principal component analysis (PCA) with factor rotation for dimension reduction to identify AD-associated principal components (PCs) and their key miRNAs with factor loadings higher than 0.8. We investigated their association with amyloid/tau/neurodegeneration (A/T/N) biomarkers and cognition. After identifying the candidate target genes of key miRNAs, we performed pathway enrichment analysis. We conducted mediation analyses to assess the effect of the associations between miRNAs and A/T/N biomarkers on AD diagnosis and cognition. Finally, we used a machine learning approach to evaluate the performance of key miRNAs for AD classification.

**Results:**

PCA identified one PC as significantly associated with AD. The PC was also significantly associated with CSF p-tau levels, hippocampal volume, and cognition. Two key miRNAs (miR-423-5p and miR-92a-3p) in the PC were associated with AD. Lower levels of miR-423-5p and miR-92a-3p were associated with reduced hippocampal volume and worse cognition, and lower levels of miR-423-5p were associated with higher brain amyloid deposition. Pathway enrichment analysis identified several significant biological processes, including memory, protein phosphorylation, and the phosphatidylinositol-3-phosphate biosynthetic process. Mediation analysis revealed that miR-423-5p, but not miR-92a-3p, had indirect effects on AD diagnosis and memory performance through brain amyloid deposition and brain atrophy. Machine learning analysis demonstrated that incorporating two key miRNAs improved the performance of demographic information for AD classification.

**Conclusion:**

Plasma miR-423-5p and miR-92a-3p are implicated in AD pathology and cognitive decline, providing insights into their roles in disease mechanisms. This study suggests the potential of these miRNAs as blood-based molecular biomarkers for AD.

## 1 Introduction

Alzheimer’s disease (AD) is a progressive neurodegenerative disorder that stands as a common cause of dementia among older adults (Long and Holtzman, 2019). AD manifests through a constellation of neuropathological features, including the extracellular accumulation of amyloid-*β* (Aβ) plaques along with intracellular neurofibrillary tangles (NFTs) composed of hyperphosphorylated tau protein aggregates (Long and Holtzman, 2019). Despite extensive research efforts, the pathogenesis of AD remains elusive, largely due to its complex neurochemical and genetic landscape (Hampel et al., 2010).

Significant advancements have been made in antemortem diagnosis of AD through the use of biomarkers obtained from cerebrospinal fluid (CSF) or visualized via positron emission tomography (PET) imaging (Long and Holtzman, 2019; Schindler et al., 2024). These biomarkers have proven to be highly effective in identifying the pathological hallmarks of the disease (Hampel et al., 2018; Long and Holtzman, 2019; Schindler et al., 2024). However, the invasive nature of CSF collection and the high costs of PET imaging pose significant limitations to their widespread application in routine clinical practice (Hampel et al., 2018; Schindler et al., 2024). Consequently, there is a critical need for the identification of more accessible and less invasive biomarkers, such as blood-based ones, for AD diagnosis.

In recent years, microRNAs (miRNAs), which are non-coding RNA molecules approximately 20–23 nucleotides long, have emerged as potential biomarkers for AD (Wang et al., 2019; Kou et al., 2020). These miRNAs are involved in AD pathogenesis, playing important roles in post-transcriptional gene expression regulation by binding the 3′-untranslated regions of messenger RNAs (mRNAs) (Bartel, 2004; Winter et al., 2009; Wang et al., 2019; Kou et al., 2020). Notably, alterations in the levels of specific miRNAs in peripheral blood have been reported in AD patients, suggesting their potential utility as non-invasive biomarkers for AD (Kou et al., 2020; Guévremont et al., 2022). Given the stability of miRNAs in circulation and their detectability in various biological fluids including plasma, they represent promising candidates for the development of blood-based diagnostic biomarkers for AD (Weber et al., 2010; Turchinovich et al., 2011).

A critical step in analyzing high-dimensional biological data, such as miRNA expression data, is dimension reduction. This step is essential for simplifying complex datasets to enhance data interpretability while retaining the biological significance of the data (Meng et al., 2016). Principal component analysis (PCA) is a widely utilized method for dimension reduction, enabling the identification of underlying patterns in data that may not be apparent in high-dimensional space (Ringnér, 2008; Meng et al., 2016). Through the application of PCA, essential features are extracted from an extensive set of miRNAs, which helps elucidate the specific roles of miRNAs in AD and deepens understanding of their dysregulated expression in the context of the disease (Ringnér, 2008; Jolliffe and Cadima, 2016; Meng et al., 2016).

In this study, we investigated the association of circulating miRNAs with central AD biomarkers and their potential as candidate blood-based molecular biomarkers for AD using expression data from 64 individually measured plasma miRNAs from 145 participants in the Alzheimer’s Disease Neuroimaging Initiative (ADNI) dataset. We performed PCA to identify AD-associated principal components (PCs) and their key miRNAs. Subsequently, we also investigated their association with amyloid/tau/neurodegeneration (A/T/N) biomarkers and cognitive function, aiming to contribute to the identification of potential blood-based molecular biomarkers for AD. After identifying the candidate target genes of these miRNAs, we investigated their involvement in biological pathways to provide insights into the molecular mechanisms underpinning AD. Furthermore, we performed mediation analysis to assess the effect of the associations between miRNAs and A/T/N biomarkers on AD diagnosis and cognitive function. Finally, we conducted a machine learning approach to evaluate the performance of miRNAs for the classification of AD.

## 2 Materials and methods

### 2.1 Study samples

The ADNI was launched in 2003 through a collaborative effort including the National Institute on Aging, National Institute of Biomedical Imaging and Bioengineering, Food and Drug Administration, private pharmaceutical companies, and nonprofit organizations. The initial phase, ADNI-1 (Petersen et al., 2010), aimed to assess the feasibility of using serial magnetic resonance imaging (MRI), PET scans, various biological markers, and clinical and neuropsychological assessments as reliable indicators of AD pathogenesis. This was followed by subsequent phases—ADNI-GO (Aisen et al., 2010), ADNI-2 (Aisen et al., 2015), and ADNI-3 (Weiner et al., 2017)—which expanded on the initial phase by allowing for the continuous follow-up of existing participants and incorporating new enrollments. Detailed information on ADNI, including latest updates, eligibility criteria, protocols for clinical and neuroimaging evaluations, and an outline of diagnostic criteria is available at https://www.adni-info.org. The ADNI Laboratory of Neuro Imaging (LONI) website^1^ provided access to demographic and clinical information, raw neuroimaging data, CSF biomarker data, apolipoprotein E (*APOE*) ε4 genotyping, and cognitive scores. All participants provided written informed consent at the time of enrollment, which included consent for data analysis and sharing. The study received approval from the Institutional Review Board at each participating site. Participants diagnosed with probable AD and cognitively normal (CN) were followed up prospectively with clinical data, neuroimaging studies, and biological samples for molecular biomarker measurements, as previously described (Aisen et al., 2010; Petersen et al., 2010; Aisen et al., 2015; Weiner et al., 2017). Diagnostic classifications were based on assessments including the Logical Memory from the Wechsler Memory Scale—Revised (Wechsler, 1987), Mini-Mental State Examination (MMSE) (Folstein et al., 1975), and Clinical Dementia Rating scale (Morris, 1993).

### 2.2 miRNA expression data

To assess concordance of miRNA expression between CSF and plasma, the same RT-qPCR workflow was applied to both biofluids using a custom TaqMan Advanced Human miRNA Low-Density Array (TLDA) card containing 64 preselected miRNAs. These miRNAs were selected based on prior literature demonstrating their detectability in CSF (Lusardi et al., 2016; Saugstad et al., 2017; Wiedrick et al., 2019; Sandau et al., 2020b; Sandau et al., 2022) and plasma (Sandau et al., 2020a) and their established relevance to AD. RNA isolation was conducted in 24-sample batches, including matched CSF and plasma samples to mitigate batch effects. Total RNA was extracted from 250 μL each of CSF and plasma using the miRNA Purification kit, following the manufacturer’s protocol (Abdalla et al., 2011). A 3 pM concentration of the exogenous spike-in cel-miR-39-3p was added following the lysis step. RNA elution was performed with 30 μL of nuclease-free water for CSF samples and 50 μL for plasma samples. Quantification of miRNA was performed using the Qubit miRNA Assay kit and Qubit 4.0 fluorometer (Scientific, 2017). For reverse transcription quantitative polymerase chain reaction, miRNA was reverse transcribed to complementary DNA (cDNA) using the TaqMan Advanced miRNA cDNA Synthesis kit according to the manufacturer’s instructions (TaqMan, 2011). This process included poly-adenylation and adaptor ligation, followed by reverse transcription, and a universal 14-cycle miRNA amplification. The diluted miRNA amplification reaction mixture was combined with TaqMan Fast Advanced master mix and then loaded onto a custom TLDA card for assay on a QuantStudio 7 Flex. Data analysis included a detailed examination of quantification cycle (Cq) values for artifacts and quality control (QC) using array card well-level statistics. For both CSF and plasma samples, Cq values were normalized using the exogenous spike-in control cel-miR-39-3p and the endogenous reference hsa-miR-16-5p (Moldovan et al., 2014). Handling of censored values (Cq > 34) was performed through censoring-aware methods, as previously described in detail (Wiedrick et al., 2019; Sandau et al., 2024).

All 64 miRNAs passed predefined QC thresholds and were retained for downstream analysis. A total of 64 miRNAs were analyzed from 80 probable AD patients and 80 CN individuals. Participants who lacked one or more miRNA expression data were excluded for subsequent PCA, resulting in 74 probable AD patients and 71 CN individuals included for further analysis.

### 2.3 Neuroimaging data

Brain MRI scans were obtained from participants using 3 T scanners, following the ADNI standardized protocol for 3D Magnetization Prepared Rapid Gradient Echo sequences. The scans were processed through the longitudinal pipeline of FreeSurfer version 5.3, as previously described (Jack et al., 2008; Jack et al., 2010), enabling the extraction of critical regions of interest, such as the bilateral hippocampal volumes and total intracranial volume.

Additionally, preprocessed [18F] florbetapir PET scans were sourced from the ADNI database via the LONI platform (see text footnote 1, respectively) and were acquired in line with the protocols established by previous studies (Jagust et al., 2010; Jagust et al., 2015). The quantification of amyloid burden was conducted by analyzing the standard uptake value ratio (SUVR) values from [18F] florbetapir PET scans, which is essential for evaluating amyloid deposits in the brain. These SUVR values were normalized to the intensity measurements from the cerebellum, which served as a reliable reference region (Joshi et al., 2012). To correct for skewness in the distribution of florbetapir PET SUVR data, a logarithmic transformation was applied.

### 2.4 CSF biomarkers

CSF samples were collected through lumbar puncture in the morning, following an overnight fast. These samples were frozen within 1 h of collection and transported to the ADNI Biomarker Core Laboratory at the University of Pennsylvania Medical Center, according to the ADNI protocol (Kang et al., 2015). Concentrations of Aβ42 and phosphorylated tau181 (p-tau) in CSF were measured using the Roche Elecsys Aβ42 and p-tau CSF immunoassays, respectively, as previously outlined (Bittner et al., 2016; Hansson et al., 2018). The CSF biomarker data were accessed and downloaded from the ADNI LONI website (see text footnote 1, respectively). To correct for data skewness, a logarithmic transformation of the CSF biomarker data was performed.

### 2.5 Comprehensive neuropsychological assessment

Domain-specific cognitive composite scores were calculated for memory and executive function. The composite score for memory integrated results from the Alzheimer’s Disease Assessment Scale-Cognitive Subscale (Mohs et al., 1997), the Rey Auditory Verbal Learning Test (Rey, 1983), the memory components of the MMSE (Folstein et al., 1975), and the Logical Memory task (Wechsler, 1987). For executive function, the composite score included assessments from the Wechsler Adult Intelligence Scale–Revised Digit Symbol Substitution task (Wechsler and De Lemos, 1981) and the Digit Span backward task (Wechsler, 1987), the Trail Making Test Parts A and B (Reitan and Wolfson, 1985), category fluency (animals and vegetables) (Morris et al., 1989), and five clock drawing tasks. To ensure comparability and consistency across individuals within the ADNI cohorts, these composite scores were standardized to a mean value of 0 and a standard deviation of 1.

### 2.6 A/T/N biomarkers

We used CSF Aβ42 levels and global cortical amyloid deposition measured from amyloid PET scans as biomarkers of Aβ (A), CSF p-tau levels as a biomarker of tau (T), and structural hippocampal atrophy on brain MRI as a biomarker of neurodegeneration (N), as described in the National Institute on Aging–Alzheimer’s Association Research Framework (Jack et al., 2024).

### 2.7 Dimension reduction in miRNA expression analysis using PCA

The procedure for PCA commenced with the extraction of PCs followed by an orthogonal rotation to maximize the variance explained by each PC, thus streamlining the data structure while preserving critical biological information (Jolliffe, 2002; Ringnér, 2008). The selection process for PCs was based on rigorous criteria, only retaining components with eigenvalues over 1. This threshold is widely accepted for ensuring the inclusion of PCs representing significant variance and relevance (Jolliffe, 2002; Peres-Neto et al., 2005). A factor loading threshold exceeding 0.8 was applied to identify the key miRNAs for each rotated PC, thereby focusing exclusively on miRNAs that have a substantial impact on the PCs. This selective approach aligns with methodologies that aim to improve data interpretability and significance (Fabrigar et al., 1999), excluding miRNAs with lower loadings from further analysis.

### 2.8 Identification of target genes for key miRNAs in AD-associated PCs

To understand the biological implications of the key miRNAs identified in the PCs associated with AD, their target genes were predicted using two well-known databases: TargetScan (Lewis et al., 2005) and miRDB (Wong and Wang, 2015). These databases are pivotal in the field of miRNA research, offering a comprehensive collection of both predicted and experimentally validated interactions that outline the relationships between miRNAs and their corresponding target genes. To ensure the robustness of our analysis, we applied a rigorous selection criterion for further analysis, focusing only on target genes consistently identified in both databases.

### 2.9 Pathway-based enrichment analysis of target genes

To explore intricate molecular pathways and biological processes implicated by the target genes of AD-associated key miRNAs, a gene-set enrichment analysis was performed using the Database for Annotation, Visualization, and Integrated Discovery (DAVID) online resource (Huang et al., 2009). DAVID is widely recognized for its comprehensive analytical framework, enabling the systematic identification of functionally related gene groups and their associated biological pathways. We specifically focused on the Gene Ontology (GO) - particularly the Biological Processes (BP) category (Ashburner et al., 2000). To adjust for multiple testing, Bonferroni correction (Dunn, 1961) was applied by dividing the significance threshold by the number of GO-BP terms tested within this specific category.

### 2.10 Statistical analysis

For the PCs of miRNAs, logistic regression models were used to evaluate the association of the PCs and key miRNAs with AD diagnosis to identify the AD-associated PCs and key miRNAs. Linear regression models were used to assess the association of the AD-associated PCs and key miRNAs with global cortical amyloid deposition, hippocampal volume, and CSF biomarkers for AD, including Aβ42 and p-tau. Moreover, linear regression models were used to investigate the association of the AD-associated PCs and key miRNAs with baseline composite scores for memory and executive function, while linear mixed effects models were used to evaluate their association with longitudinal changes in the composite scores of memory and executive function. In all association analyses, age and sex were consistently included as covariates. Additional covariates included years of education for cognitive performance, *APOE* ε4 carrier status for global cortical amyloid deposition, CSF Aβ42, and CSF p-tau, and intracranial volume for hippocampal volume. For the association analysis of PCs with AD diagnosis, the false discovery rate (FDR) correction with the Benjamini-Hochberg procedure was used to adjust for multiple testing (Benjamini and Hochberg, 1995).

To assess the potential modifying effects of sex on the associations between miRNAs and A/T/N biomarkers, we conducted stratified linear regression analyses separately in males and females. The stratification variable sex was not included as a covariate in the sex-stratified analyses. Covariate-adjusted regression coefficients were transformed into sample size-weighted standardized effect sizes (Cohen’s d) to formally compare the strength of associations between subgroups (Paternoster et al., 1998). A two-sided *p*-value was calculated from the t-statistic using a standard normal distribution to assess sex-specific differences in A/T/N biomarkers based on standardized effect sizes. Additionally, we quantified the magnitude of effect heterogeneity between subgroups using the I^2^ statistic (Higgins and Thompson, 2002). An I^2^ value above 50% was considered indicative of substantial heterogeneity. To complement the subgroup difference analyses, we also included an interaction term between sex and miRNA in the pooled linear regression model. The *p* value for the interaction term was calculated to evaluate whether the association between each miRNA and A/T/N biomarkers significantly differed between male and female subgroups. In accordance with previously established methodology (Arnold et al., 2020), we classified miRNA-A/T/N biomarker associations into homogeneous effects if miRNAs showed very similar effects in their association to the biomarker for both sexes (i.e., estimated heterogeneity *p* > 0.05), and heterogeneous effects if miRNAs showed different effects in both sexes leading to significant heterogeneity (i.e., estimated heterogeneity *p* < 0.05) and/or the interaction between sex and miRNA. Effects that were significant in only one sex with either significant effect heterogeneity between males and females or significant interaction between sex and miRNA were considered sex-specific.

For the key miRNAs, we performed Pearson correlation analyses of their expression levels between plasma and CSF in participants with paired biofluid samples.

We performed mediation analysis on the key miRNAs demonstrating a significant correlation with AD diagnosis and a composite score of memory, following established methodologies (Hayes, 2009). Our investigation aimed to determine whether the identified relationships could be mediated by biomarkers for AD. For these analyses, we used the Mediation R package, which allowed for the evaluation of indirect effects through a bootstrapping approach (Tingley et al., 2014).

To investigate the classification performance of AD-associated key miRNAs in distinguishing AD patients from CN individuals, we applied the STREAMLINE tool (Urbanowicz et al., 2023), using an extreme gradient boosting machine learning approach. This machine learning approach is selected for integrating multi-view biological data and heterogeneous feature sets for the analysis of complex biological systems (Li et al., 2018). The dataset was randomly partitioned into 70% for training the model and 30% for testing, with a 5-fold cross-validation process to enhance the robustness and reliability of model evaluation. The classification performance of two different models in differentiating AD from CN was assessed through the receiver operating characteristic (ROC) curve and the area under the receiver operating characteristic curve (AUC). In terms of feature selection, Model 1 included age, sex, and *APOE* ε4 carrier status, while Model 2 incorporated all features from Model 1 and additionally included AD-associated key miRNAs, aiming to investigate their additional classification performance values in distinguishing AD from CN.

**Figure 1:**
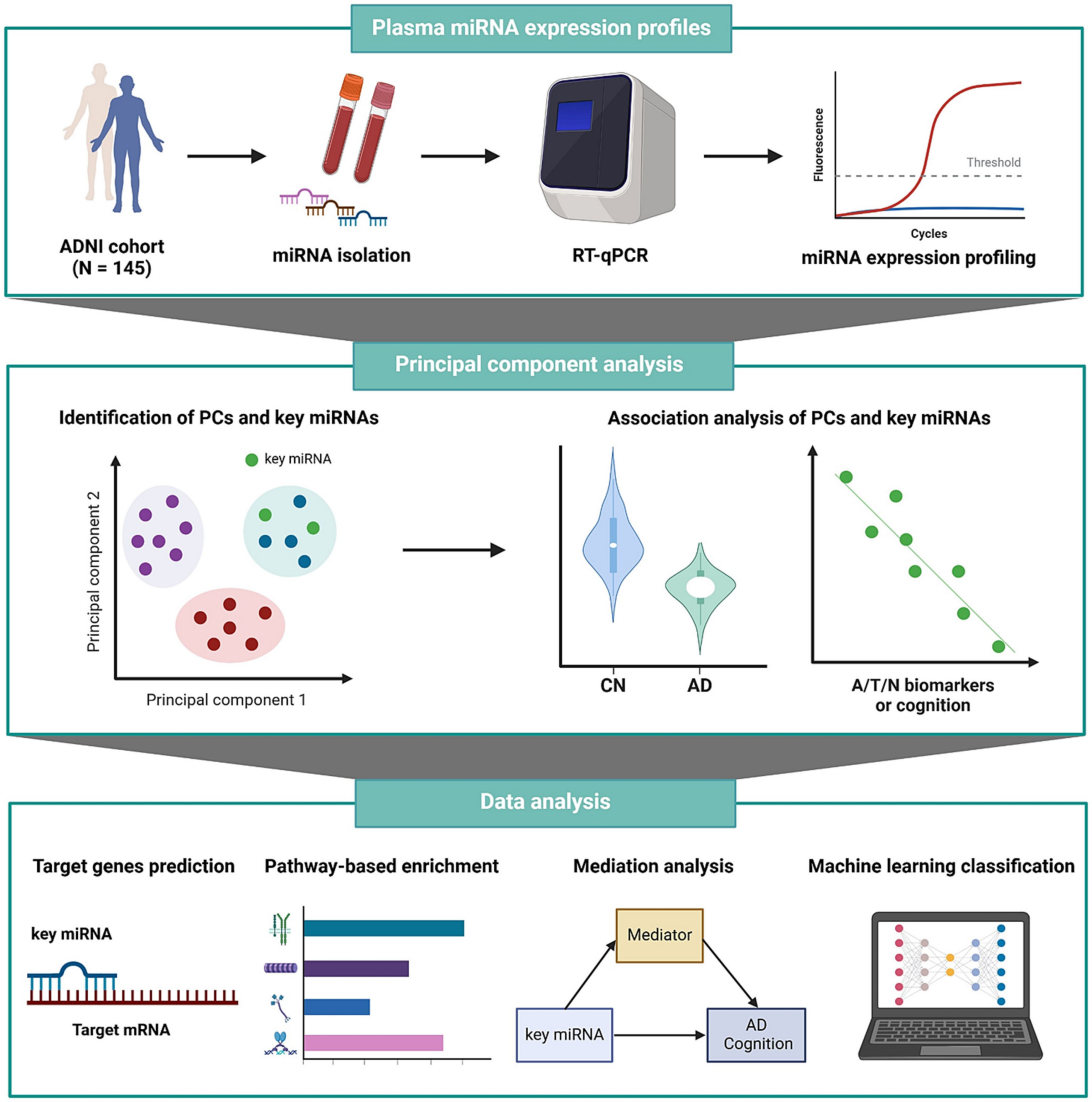
Schematic overview of the workflow of our analysis. A/T/N, amyloid/tau/neurodegeneration; AD, Alzheimer’s disease; ADNI, Alzheimer’s Disease Neuroimaging Initiative; CN, cognitively normal; miRNA, microRNA; mRNA, messenger RNA; PC, principal component; RT-qPCR, reverse transcription quantitative polymerase chain reaction.

All statistical analyses were performed using R software, version 4.2.2. We defined statistical significance at a *p* value threshold of less than 0.05, incorporating adjustments for multiple comparisons. The workflow of all analysis steps used in this study is shown in Figure 1.

## 3 Results

### 3.1 Demographic and clinical characteristics

We used miRNA expression data from 145 ADNI participants, including 74 probable AD patients and 71 CN individuals, as detailed in Table 1. The median age was 74.5 years, with 56.3% being females. No significant differences were observed in sex distribution and age across the diagnostic groups. However, a substantial difference in *APOE* ε4 carrier status was noted, with a higher prevalence in AD patients compared to CN individuals. For the A/T/N biomarkers, CSF A*β*42 and hippocampal volume were significantly lower, and CSF p-tau levels and global cortical amyloid deposition were significantly higher in AD patients compared to CN individuals. Furthermore, composite scores for memory and executive function were significantly lower in AD patients compared to CN individuals.

**Table 1:** Demographic information of participants from the ADNI cohort.

	CN (*n* = 71)	AD (*n* = 74)	Total (*n* = 145)	*p* value^b^
Male (%)	36 (50.7)	44 (59.5)	80 (55.2)	0.289
Age, years^a^	73.7 (69.7–78.8)	75.7 (69.1–80.7)	74.5 (69.6–79.7)	0.335
Education, years^a^	16 (14–18)	16 (13–16)	16 (14–18)	0.011
*APOE* ε4 carrier (%)	21 (29.6)	50 (67.6)	71 (49.0)	<0.001
CSF Aβ42, pg/mL^a^	1255.5 (826.5–1800.5)	650.2 (482.7–814.0)	809.7 (577.6–1411.0)	<0.001
CSF p-tau, pg/mL^a^	19.2 (16.7–24.2)	33.6 (27.4–47.5)	26.5 (18.7–36.7)	<0.001
Amyloid PET global SUVR^a^	1.04 (0.98–1.19)	1.46 (1.32–1.58)	1.24 (1.03–1.47)	<0.001
Hippocampal volume, mm^3a^	3,753 (3,520 – 4,000)	3,020 (2,794 – 3,266)	3,385 (2,974 – 3,777)	<0.001
Intracranial volume, mm^3a^	1,481,766 (1,366,350 – 1,633,833)	1,458,122 (1,371,248 – 1,592,867)	1,469,878 (1,369,858 – 1,629,653)	0.899
Baseline memory, Z scores^a^	0.93 (0.56–1.23)	−0.75 (−1.00 – −0.58)	−0.07 (−0.75–0.88)	<0.001
Baseline executive function, Z scores^a^	0.68 (0.42–1.07)	−0.39 (−0.97–0.07)	0.24 (−0.46–0.73)	<0.001

Values are *n* (%), unless indicated otherwise. ^a^Data are presented as median followed by (interquartile range). ^b^The Mann–Whitney U test or chi-square test was used to determine the p value for comparisons between AD and CN groups, as appropriate. Aβ, amyloid-β; AD, Alzheimer’s disease; ADNI, Alzheimer’s Disease Neuroimaging Initiative; APOE, apolipoprotein E; CN, cognitively normal; CSF, cerebrospinal fluid; p-tau, phosphorylated tau; PET, positron emission tomography; SUVR, standard uptake value ratio.

### 3.2 Principal component analysis for dimension reduction of miRNA expression data

PCA was performed on 64 miRNAs in 74 probable AD patients and 71 CN individuals from the ADNI cohort. Dimension reduction with PCA resulted in 13 PCs with eigenvalues > 1 (Supplementary Table S1). After selecting key miRNAs with a factor loading ≥ 0.8 in each principal component, 7 of 13 PCs remained for further analysis (Supplementary Table S2).

**Figure 2:**
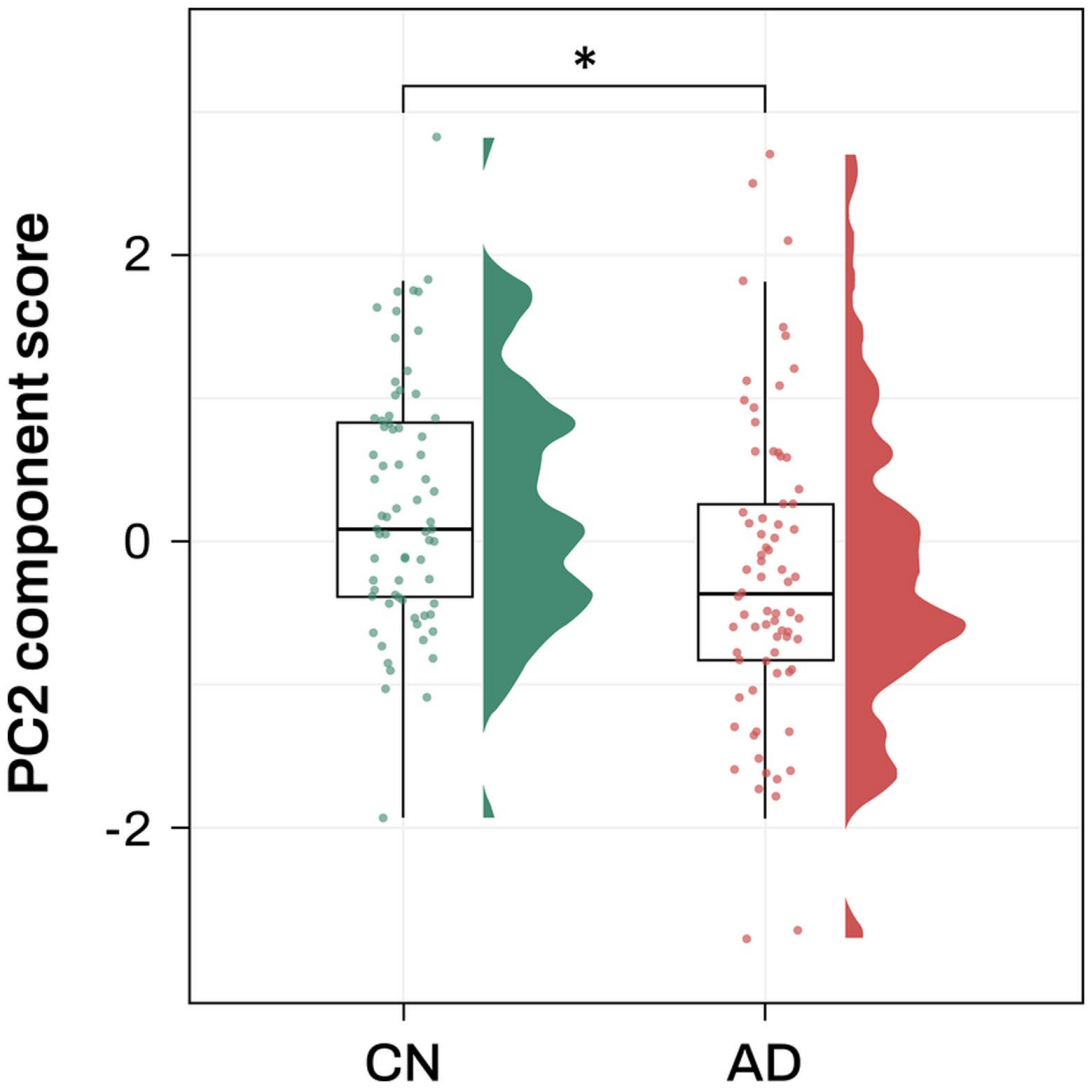
Association analysis results of PC2 with AD diagnosis Violin and box plots represent the PC2 component score for AD diagnosis. AD, Alzheimer’s disease; CN, cognitively normal; PC, principal component. Note: significance stars indicating the *p* values of the correlations * *p*-value < 0.05.

Association analysis of the 7 PCs with AD diagnosis identified PC2 as significantly associated with AD after multiple comparison correction (odds ratio (OR) = 0.59, FDR-corrected *p* = 0.038; Figure 2; Supplementary Table S3).

### 3.3 Association of AD-associated PC2 with A/T/N biomarkers

**Figure 3:**
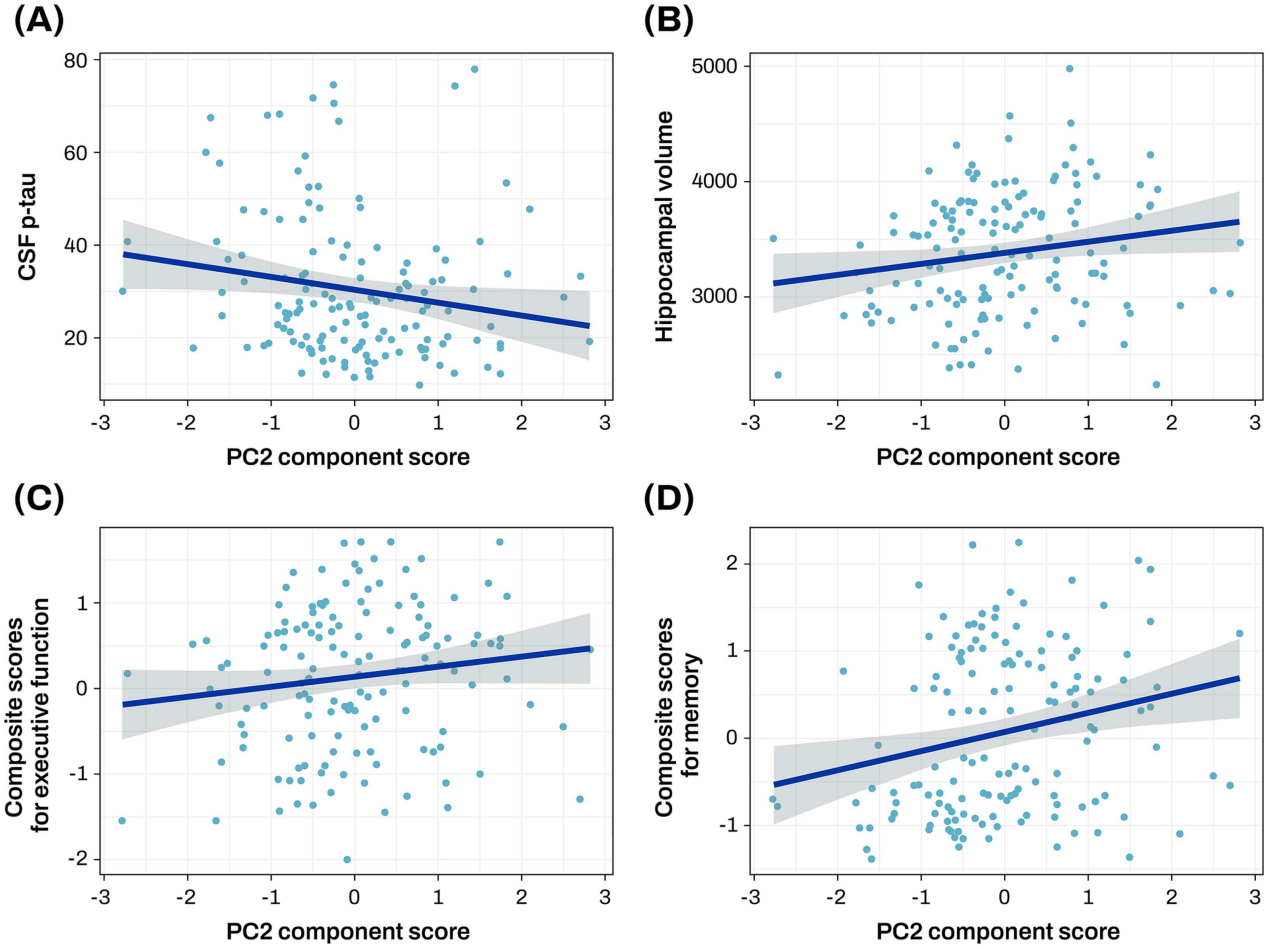
Association analysis results of PC2 with CSF p-tau, hippocampal volume, and cognitive function The scatter plot represents a correlation of the PC2 component score with CSF p-tau **(A)**, hippocampal volume **(B)**, baseline composite score for executive function **(C)**, and baseline composite score for memory **(D)**. The gray zone around the linear regression line represents the 95% confidence interval. Hippocampal volume was measured in cubic millimeters (mm^3^). CSF biomarker concentrations were log-transformed and reported in picograms per milliliter (pg/mL). Cognitive scores were standardized as Z-scores. AD, Alzheimer’s disease; CN, cognitively normal; CSF, cerebrospinal fluid; p-tau, phosphorylated tau; PC, principal component.

The PC2 was investigated to assess its associations with central A/T/N biomarkers for AD. Lower PC2 scores were significantly associated with higher levels of CSF p-tau (*β* = −0.035 ± 0.016, *p* = 0.033) and reduced hippocampal volume (*β* = 90.105 ± 39.647, *p* = 0.025; Figures 3A,B; Supplementary Table S4).

### 3.4 Association of AD-associated PC2 with cognition at baseline and longitudinal change of cognition

**Figure 4:**
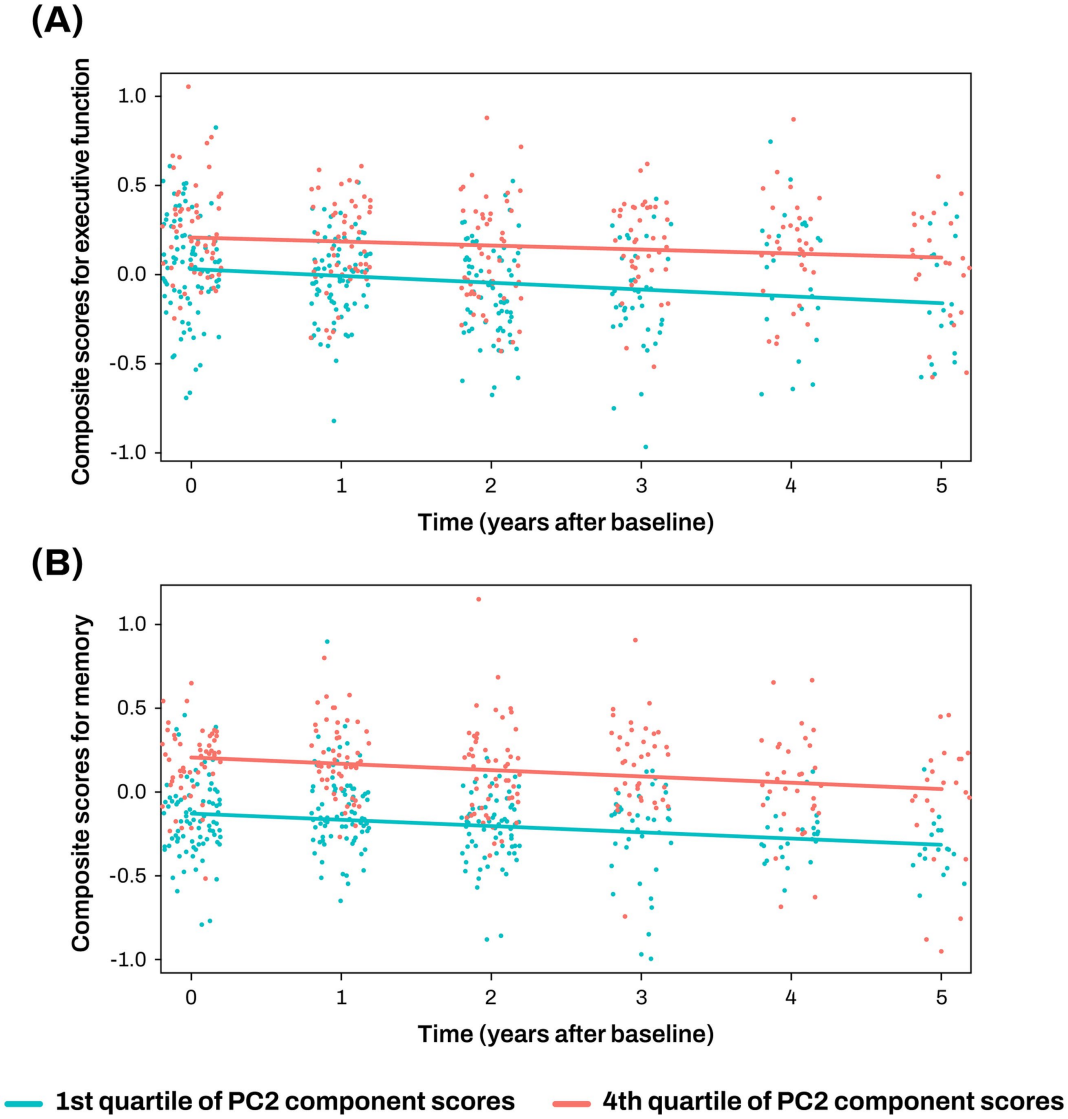
Association analysis results of PC2 with longitudinal changes of cognition **(A, B)** The x-axis represents years after the initial clinical evaluation, while the y-axis indicates composite scores for executive function **(A)** and memory **(B)**. The two lines represent the different slopes of changes in composite scores for subjects with 1st quartile (green color) versus 4th quartile (red color) stratified by PC2 component score. Cognitive scores were standardized as Z-scores. PC, principal component.

The PC2 was investigated to assess its associations with composite scores for memory and executive function at baseline and longitudinal changes of composite scores for memory and executive function. Higher PC2 scores were associated with better baseline composite scores for memory (*β* = 0.222 ± 0.074, *p* = 0.003) and executive function (*β* = 0.135 ± 0.064, *p* = 0.038; Figures 3C,D; Supplementary Table S5), and slower longitudinal decline of composite scores for memory (*β* = 0.234 ± 0.086, *p* = 0.008; Figure 4; Supplementary Table S5).

### 3.5 Association of key miRNAs in the AD-associated PC2 with AD diagnosis and A/T/N biomarkers

**Figure 5:**
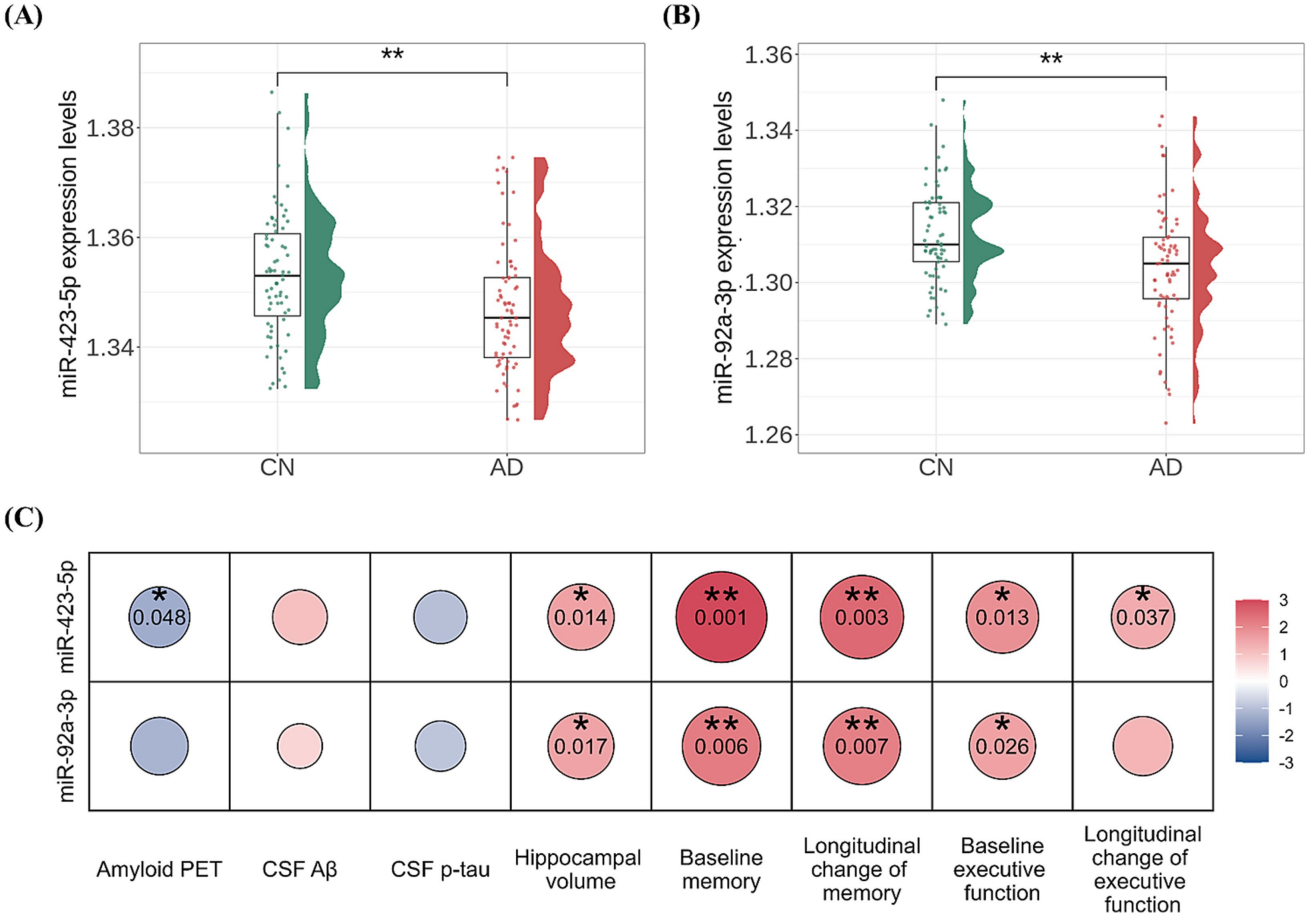
Association analysis results of miR-423-5p and miR-92a-3p with amyloid, tau, and neurodegeneration (A/T/N) biomarkers as well as cognition **(A, B)** Violin and box plots represent the miR-423-5p **(A)** and miR-92a-3p expression levels **(B)** for AD diagnosis **(C)**. Heatmap of association of miR-423-5p and miR-92a-3p expression levels with the “A/T/N” biomarkers for AD and cognition. *p* values were estimated from linear regression analyses. Circle color indicates the direction of the regression coefficient (*β*), while circle size and color intensity reflect the significance level of the association, quantified as –log (*p* value). The color bar represents –log (*p* value) × Sign(β). Red colors represent positive associations, and blue colors denote negative associations. The darker the color and larger the circle, the stronger the association. Hippocampal volume was measured in cubic millimeters (mm^3^). CSF biomarker concentrations were log-transformed and reported in picograms per milliliter (pg/mL). Cognitive scores were standardized as Z-scores. Aβ, amyloid-β; AD, Alzheimer’s disease; CN, cognitively normal; CSF, cerebrospinal fluid; miR, microRNA; p-tau, phosphorylated tau; PET, positron emission tomography. Note: significance stars indicating the *p* values of the correlations adjustment for multiple comparisons * *p*-value < 0.05. ** *p*-value < 0.01.

**Figure 6:**
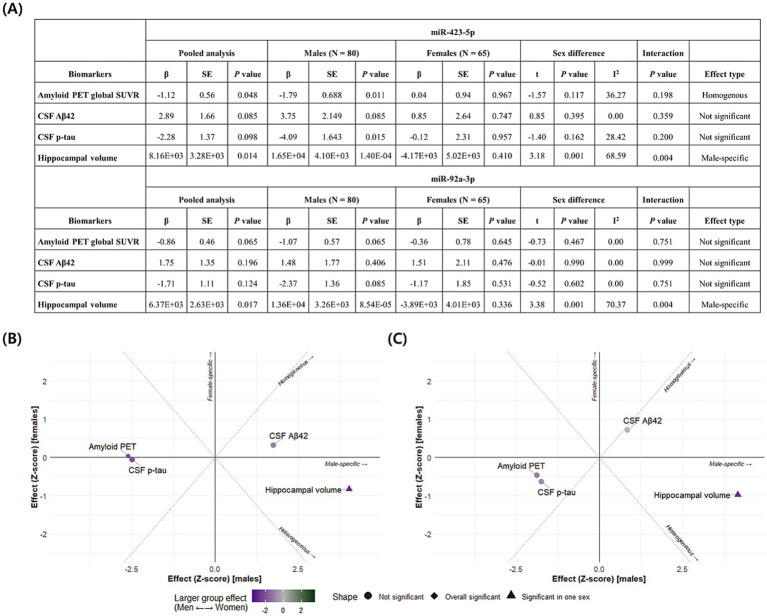
Association analysis results and sex-based effect heterogeneity of miR-423-5p and miR-92a-3p with amyloid, tau, and neurodegeneration (A/T/N) biomarkers **(A)** Linear regression results examining the associations between miRNAs and A/T/N biomarkers are presented for the full sample and stratified by sex, along with estimates of effect heterogeneity and *p* value for sex-by-miRNA interaction terms. Scatter plots display Z-scores of effect estimates for the associations of the miR-423-5p **(B)** and miR-92a-3p **(C)** with A/T/N biomarkers in males (x-axis) and females (y-axis). Homogeneous effects, defined as associations with comparable effect size in both sexes, appear along the diagonal. Heterogeneous effects appear along the anti-diagonal. Sex-specific effects are located near the x-axis (male-specific) or y-axis (female-specific effects). Homogeneous and overall significant results are indicated by diamonds, while effects significant in only one sex are represented as triangles. Sex-specific effects are further visualized using a color scale (purple for males; green for females). Hippocampal volume was measured in cubic millimeters (mm^3^). CSF biomarker concentrations were log-transformed and reported in picograms per milliliter (pg/mL). Aβ, amyloid-β; CSF, cerebrospinal fluid; miR, microRNA; p-tau, phosphorylated tau; PET, positron emission tomography; SE, Standard Error; SUVR, standardized uptake value ratio.

In the AD-associated PC2, two miRNAs, miR-423-5p and miR-92a-3p, with a factor loading higher than 0.8 were identified as key miRNAs (Supplementary Table S2). Further analyses were performed to investigate the association of these two key miRNAs with AD diagnosis and A/T/N biomarkers for AD. Expression levels of miR-423-5p and miR-92a-3p were significantly lower in AD patients compared to CN individuals (miR-423-5p, *β* = −44.438 ± 15.286, *p* = 0.004; miR-92a-3p, *β* = −41.606 ± 13.018, *p* = 0.001; Figures 5A,B). Lower expression levels of miR-423-5p and miR-92a-3p were significantly associated with reduced hippocampal volume (miR-423-5p, *β* = 8.162 × 10^3^ ± 3.279 × 10^3^, *p* = 0.014; miR-92a-3p, *β* = 6.366 × 10^3^ ± 2.632 × 10^3^, *p* = 0.017), and lower expression levels of miR-423-5p were associated with higher global cortical amyloid deposition (*β* = −1.118 ± 0.561, *p* = 0.048; Figures 5C, 6A).

We conducted sex-stratified association analyses and subsequently assessed heterogeneity in effect estimates between males and females. Figure 6 details the results of miRNA associations with A/T/N biomarkers, including sex-by-miRNA interaction analyses. We identified a significant homogeneous association between miR-423-5p and global cortical amyloid deposition. In contrast, both miR-423-5p and miR-92a-3p exhibited male-specific associations with reduced hippocampal volume.

### 3.6 Association of key miRNAs in the AD-associated PC2 with cognition at baseline and longitudinal change of cognition

**Figure 7:**
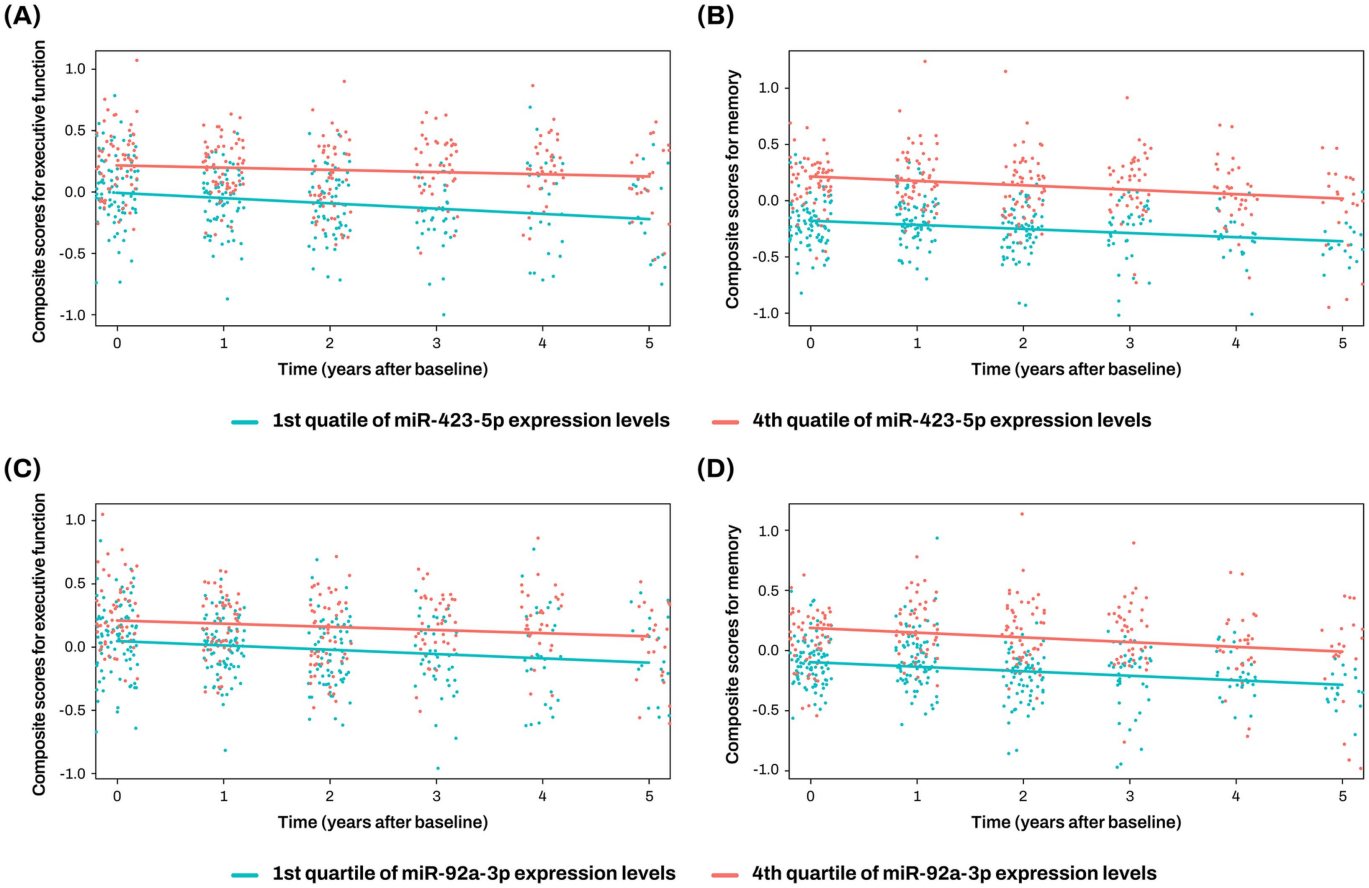
Association analysis results of miR-423-5p and miR-92a-3p with longitudinal changes of cognition The x-axis represents years after the initial clinical evaluation, while the y-axis indicates composite scores for executive function **(A,C)** and memory **(B,D)**. The two lines represent the different slopes of changes in composite scores for subjects with 1st quartile (green color) versus 4th quartile (red color) stratified by miR-423-5p **(A,B)** and miR-92a-3p expression levels **(C,D)**. Cognitive scores were standardized as Z-scores. miR, microRNA.

The two key miRNAs, miR-423-5p and miR-92a-3p, were investigated to assess their associations with composite scores for memory and executive function at baseline and longitudinal changes of composite scores for memory and executive function. Higher expression levels of miR-423-5p and miR-92a-3p were significantly associated with better baseline composite scores for memory (miR-423-5p, *β* = 20.164 ± 6.098, *p* = 0.001; miR-92a-3p, *β* = 13.652 ± 4.941, *p* = 0.006) and executive function (miR-423-5p, *β* = 13.348 ± 5.317, *p* = 0.013; miR-92a-3p, *β* = 9.624 ± 4.280, *p* = 0.026; Figure 5C; Supplementary Table S6). Higher expression levels of miR-423-5p and miR-92a-3p were also significantly associated with slower longitudinal decline of composite scores for memory (miR-423-5p, *β* = 21.573 ± 7.133, *p* = 0.003; miR-92a-3p, *β* = 15.684 ± 5.775, *p* = 0.007), and lower expression levels of miR-423-5p were associated with slower longitudinal decline of composite scores for executive function (*β* = 12.483 ± 5.927, *p* = 0.037; Figures 5C, 7; Supplementary Table S6).

### 3.7 Correlation between miRNA expression in plasma and CSF

**Figure 8:**
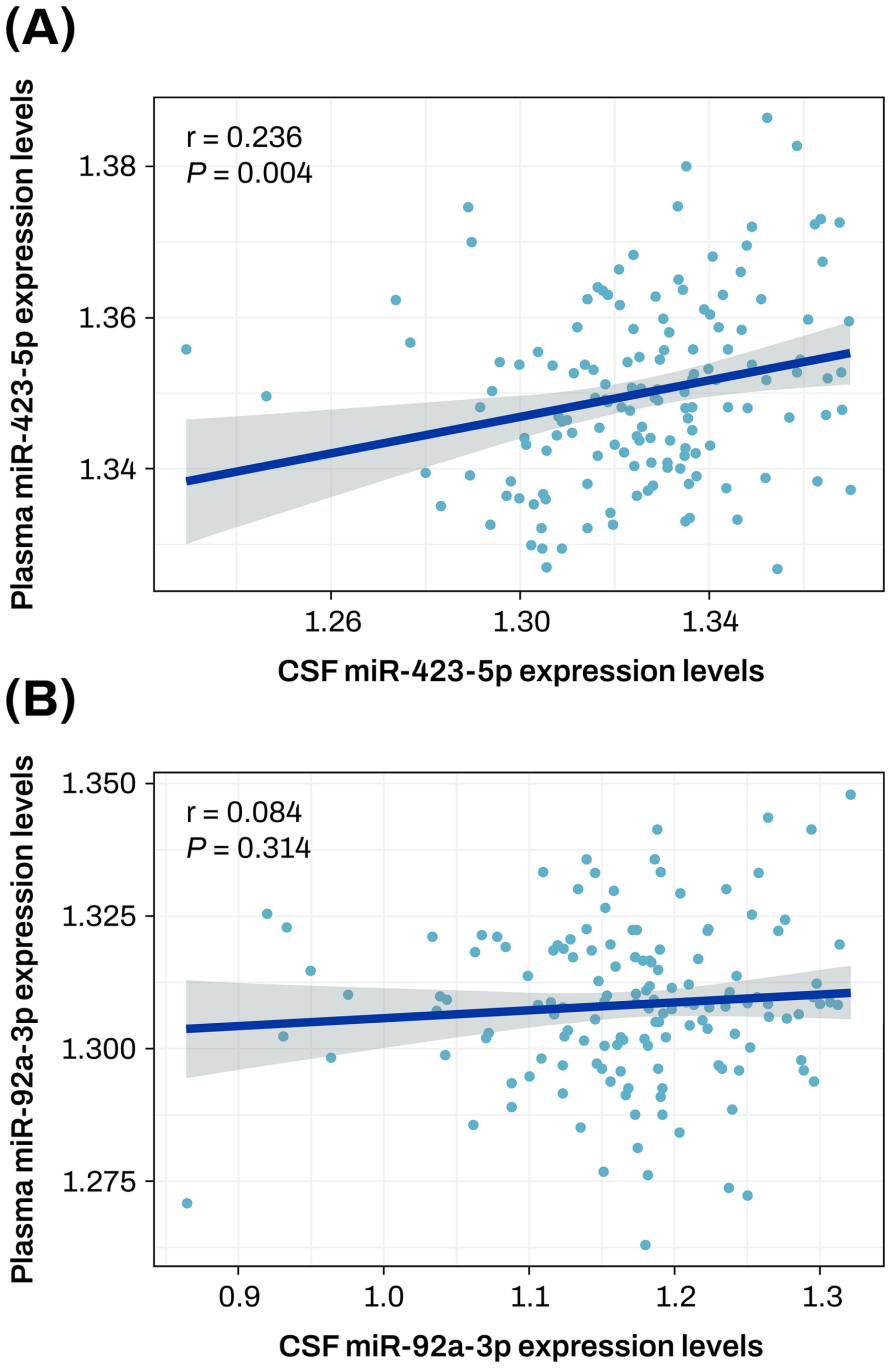
Correlation plot between plasma and CSF expression levels of miR-423-5p and miR-92a-3p **(A,B)** The scatter plot represents a correlation between plasma and CSF expression levels of miR-423-5p **(A)** and miR-92a-3p **(B)**. The gray zone around the linear regression line represents the 95% confidence interval. CSF, cerebrospinal fluid; miR, microRNA.

The two key miRNAs, miR-423-5p and miR-92a-3p, were further investigated to evaluate their correlation between plasma and CSF samples. The correlation analysis between plasma and CSF expression levels of miR-423-5p showed a significant correlation (r = 0.236, *p* = 0.004), while the significant correlation was not observed with miR-92a-3p (Figure 8).

### 3.8 Predicted target genes for two key miRNAs and their pathway enrichment analysis

Target genes for the two miRNAs, miR-423-5p and miR-92a-3p, were obtained from the miRDB and TargetScan databases, resulting in 700 and 985 genes from the miRDB database, and 232 and 1,041 genes from the TargetScan database, respectively. 106 and 701 overlapping target genes for miR-423-5p and miR-92a-3p were then identified between the two databases (Supplementary Additional file). The GO analysis of the target genes for miR-423-5p and miR-92a-3p revealed significant enrichment in BP pathway related to positive regulation of transcription from RNA polymerase II promoter, negative regulation of transcription from RNA polymerase II promoter, brain development, protein phosphorylation, intracellular signal transduction, negative regulation of transcription, peptidyl-serine phosphorylation, nervous system development, neuron migration, memory, palate development, negative regulation of cytoplasmic translation, ventricular septum morphogenesis, cell migration, peptidyl-threonine phosphorylation, positive regulation of transcription, regulation of transcription from RNA polymerase II promoter, phosphatidylinositol-3-phosphate biosynthetic process, and cellular response to amino acid stimulus (Table 2).

**Table 2:** Gene ontology analysis of target genes of miR-423-5p and miR-92a-3p.

Biological process	Number of genes from study data/Number of genes in the pathway (Population Hits)	Fold enrichment	*p* value^a^
Positive regulation of transcription from RNA polymerase II promoter	98/1232	2.088	<0.001
Negative regulation of transcription from RNA polymerase II promoter	78/1016	2.015	<0.001
Brain development	31/264	3.083	<0.001
Protein phosphorylation	40/444	2.365	<0.001
Intracellular signal transduction	41/462	2.330	<0.001
Negative regulation of transcription, DNA-templated	48/591	2.132	<0.001
Peptidyl-serine phosphorylation	23/184	3.282	<0.001
Nervous system development	37/425	2.285	0.002
Neuron migration	17/124	3.599	0.006
Memory	14/89	4.130	0.010
Palate development	12/66	4.773	0.012
Negative regulation of cytoplasmic translation	6/12	13.126	0.017
Ventricular septum morphogenesis	9/37	6.386	0.020
Cell migration	26/278	2.455	0.022
Peptidyl-threonine phosphorylation	12/72	4.375	0.027
Positive regulation of transcription, DNA-templated	50/728	1.803	0.027
Regulation of transcription from RNA polymerase II promoter	97/1736	1.467	0.039
Phosphatidylinositol-3-phosphate biosynthetic process	7/22	8.353	0.044
Cellular response to amino acid stimulus	11/64	4.512	0.047

^a^Adjusted P value for Bonferroni correction. AD, Alzheimer’s dementia; miRNAs, microRNAs; mRNA, messenger RNAs.

### 3.9 Mediation analysis of two key miRNAs on AD or composite scores for memory

**Figure 9:**
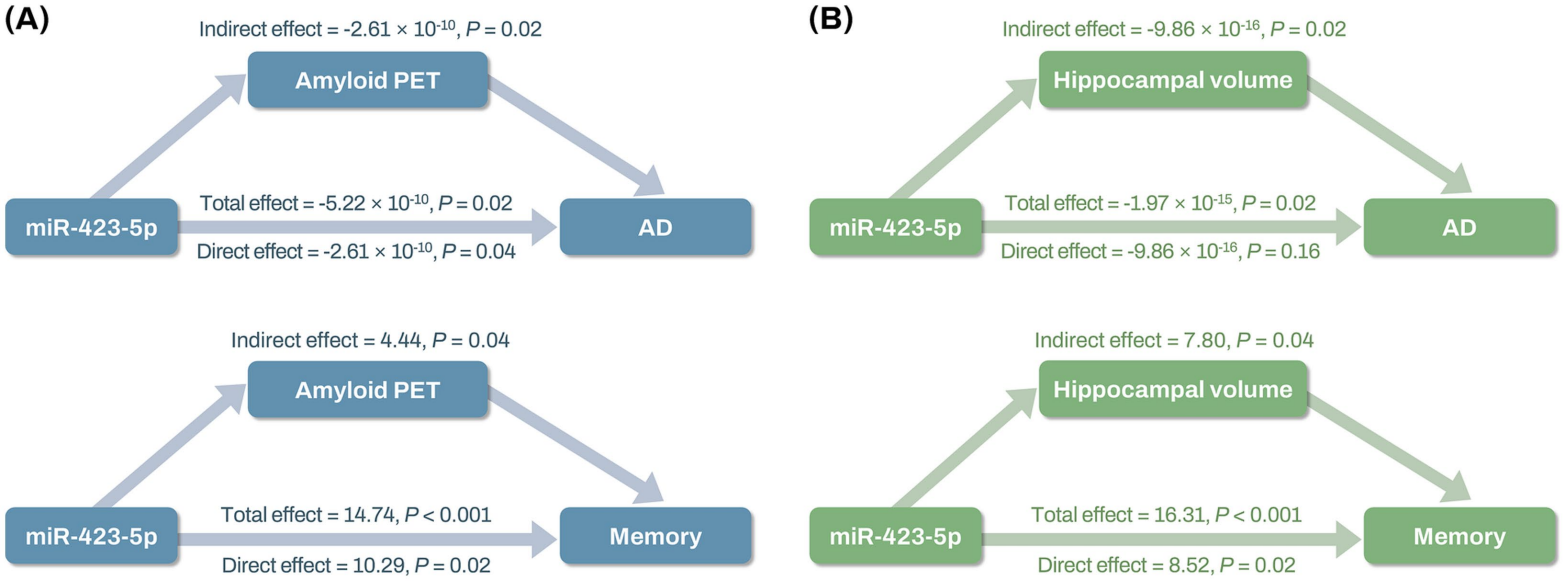
Mediation analysis results of miR-423-5p on AD diagnosis and composite scores for memory through global cortical amyloid deposition and hippocampal volume **(A,B)** Mediation analysis shows total, direct, and indirect effects of miR-423-5p expression levels on AD and composite scores for memory through global cortical amyloid deposition **(A)**, and hippocampal volume **(B)**. Hippocampal volume was measured in cubic millimeters (mm^3^). Cognitive scores were standardized as Z-scores. AD, Alzheimer’s disease; miR, microRNA; PET, positron emission tomography; SUVR, standard uptake value ratio.

Mediation analysis revealed that miR-423-5p expression levels had both direct and indirect effects on AD through global cortical amyloid deposition (direct effect: *β* = −2.61 × 10^−10^; *p* = 0.04; indirect effect: *β* = −2.61 × 10^−10^; *p* = 0.02), while miR-423-5p expression levels had only indirect effects on AD through hippocampal volume (direct effect: *β* = −9.86 × 10^−16^; *p* = 0.16; indirect effect: *β* = −9.86 × 10^−16^; *p* = 0.02; Figure 9; Supplementary Table S7). Additionally, miR-423-5p expression levels were found to affect composite scores for memory through both direct and indirect effects mediated by global cortical amyloid deposition (direct effect: *β* = 10.29; *p* = 0.02; indirect effect: *β* = 4.44; *p* = 0.04) and hippocampal volume (direct effect: *β* = 8.52; *p* = 0.02; indirect effect: *β* = 7.80; *p* = 0.02) (Figure 9; Supplementary Table S7). However, miR-92a-3p expression levels had no indirect effects on AD or composite scores for memory through hippocampal volume (Supplementary Table S7).

### 3.10 Machine learning analysis for AD classification

**Figure 10:**
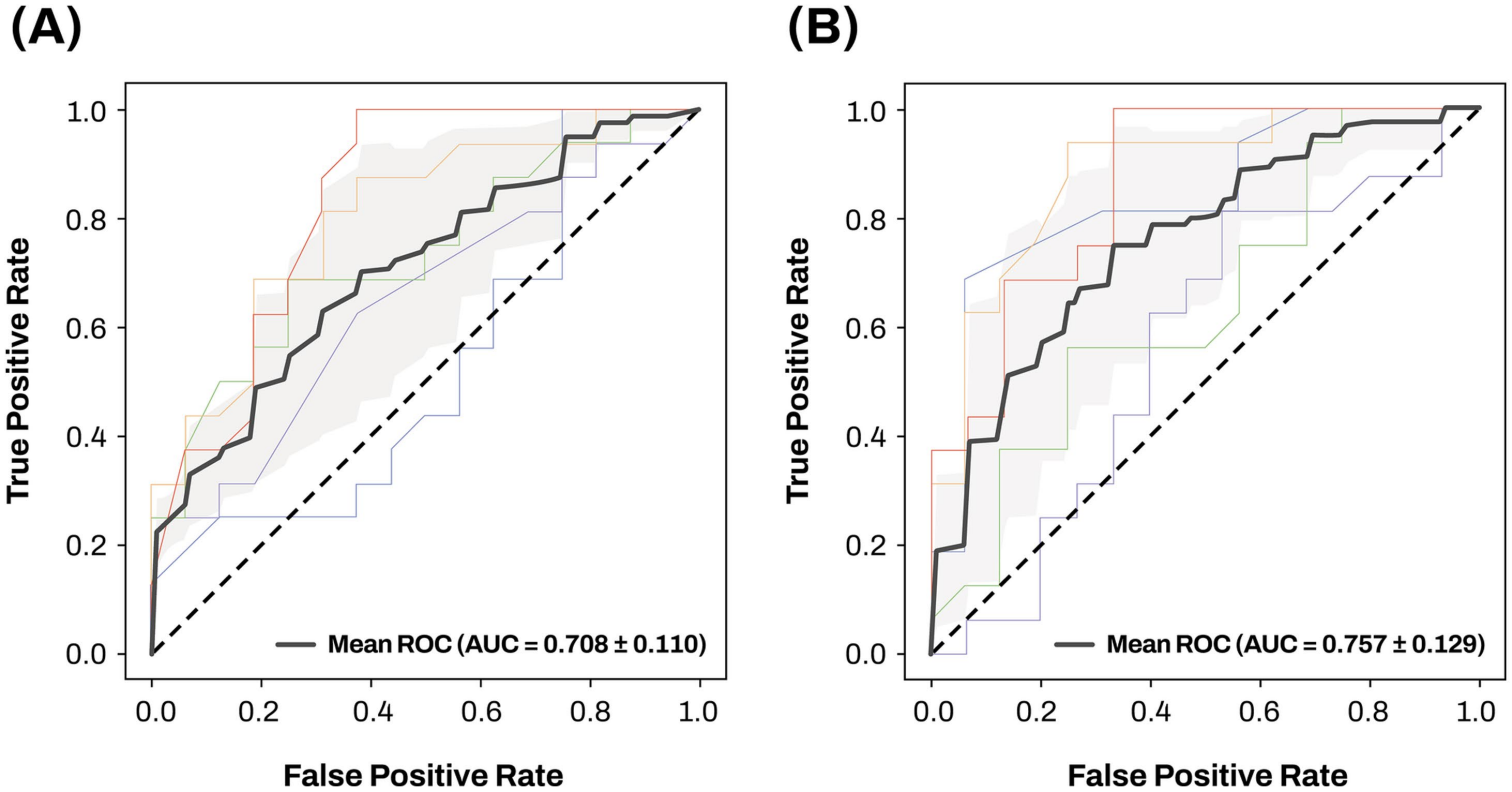
The ROC curves and mean AUC of machine learning approach Sensitivity is on the y-axis and 1-specificity is on the x-axis. 5-fold cross validation was used to investigate and compare the classification performance of two different classification models for differentiating AD from CN. The gray zone around the mean ROC curve represents ± 1 standard deviation. Two different classification models were evaluated using the following training features: **(A)** age, sex, and *APOE* ε4 carrier status; **(B)** age, sex, *APOE* ε4 carrier status, miR-423-5p, and miR-92a-3p. AD, Alzheimer’s disease; *APOE*, apolipoprotein E; AUC, area under the curve; CN, cognitively normal; miRNAs, microRNAs, ROC, receiver operating characteristic.

A machine learning approach, extreme gradient boosting, for the classification of AD patients from CN individuals was used to evaluate two different classification models. Results of the 5-fold cross-validation are presented in Figure 10. Model 1, including age, sex, and *APOE* ε4 carrier status, achieved a mean AUC value of 0.708. The mean AUC value of Model 2, obtained by adding two AD-associated key miRNAs, miR-423-5p and miR-92a-3p, to Model 1, increased to 0.757.

## 4 Discussion

This study highlights the association of circulating miRNAs with central biomarkers for AD and their potential utility as blood-based molecular biomarkers for AD. Using PCA, we identified one PC that showed significant associations with AD, CSF p-tau levels, hippocampal volume, and both baseline levels and longitudinal changes in cognition. Among the miRNAs that contributed significantly to the PC, miR-423-5p and miR-92a-3p were identified as key miRNAs. Both miRNAs were found to be significantly downregulated in AD patients compared to CN individuals, and their lower expression levels were associated with central biomarkers of AD, including reduced hippocampal volume and higher global cortical amyloid deposition. Furthermore, lower levels of these miRNAs were correlated with worse cognitive performance and a faster rate of cognitive decline, indicating their potential role not only as diagnostic biomarkers but also as indicators of disease progression.

Accumulating evidence indicates that miRNAs play important roles in AD pathogenesis through the regulation of genes involved in amyloid processing, tau phosphorylation, and neurodegeneration (Wang et al., 2019; Kou et al., 2020; Guévremont et al., 2022). Our findings align with this evidence by demonstrating associations of lower plasma levels of miR-423-5p and miR-92a-3p with higher global cortical amyloid deposition, reduced hippocampal volume, and worse cognitive performance. Moreover, our study showed a significant correlation between plasma and CSF expression levels of miR-423-5p, but not for miR-92a-3p. This suggests that miR-423-5p may have potential as a blood-based molecular biomarker reflective of central nervous system changes.

Lower expression levels of miR-423-5p were associated with AD in human cerebral white matter (Wang et al., 2011; Bhattacharyya and Bandyopadhyay, 2013), aligning with findings of our study. Importantly, our study uniquely demonstrates an association between plasma miR-423-5p levels and AD and cognitive function, which has not been previously reported. Furthermore, overexpression of miR-423-5p has been found to enhance cell proliferation, migration, and angiogenesis, and to reduce apoptosis *in vivo* (Li S. et al., 2017). Lower expression levels of plasma miR-92a-3p have been associated with AD (Peña-Bautista et al., 2022; Piscopo et al., 2023), supporting the findings of our study. Higher expression levels of plasma miR-92a-3p were also found to be significantly associated with better cognitive function (Yaqub et al., 2023; Melas et al., 2024), reinforcing the results of our study. Notably, miR-92a-3p is involved in maintaining blood–brain barrier integrity by regulating tight junctions and endothelial cell function, often compromised in AD (Lu et al., 2021). Additionally, miR-92a plays a critical role in regulating Aβ clearance, accumulation of tau protein, synaptic and gamma-aminobutyric acid-ergic dysfunction in AD (Li X. et al., 2017; Siedlecki-Wullich et al., 2019; Peña-Bautista et al., 2022). Overexpression of miR-92a-3p, one of the miRNAs identified to interact with microtubule-associated protein tau mRNAs, significantly reduced tau protein levels in neuroblastoma cell lines (Piscopo et al., 2023). We observed significant effect heterogeneity between males and females for the key miRNAs, miR-423-5p and miR-92a-3p, indicating that sex may influence miRNA-related hippocampal atrophy. Our results demonstrate the importance of stratified analyses in uncovering AD-related neurodegeneration that appear to be male-specific.

Pathway enrichment analyses conducted in this study provided insights into the significant biological processes that may be influenced by the target genes of the two AD-associated key miRNAs. Notably, dysregulation in cell migration, proliferation, and differentiation plays a significant role in both AD and Parkinson’s disease, underscoring the importance of these pathways in neurodegeneration (Awuson-David et al., 2023). Furthermore, pathways such as protein phosphorylation, including peptidyl-serine and peptidyl-threonine phosphorylation, are critical, as hyperphosphorylation of tau at these residues contributes to its aggregation into NFTs, a hallmark of AD pathology (Wegmann et al., 2021). Lastly, a deficiency in phosphatidylinositol-3-phosphate was identified as a contributing factor to AD pathogenesis by disrupting amyloid precursor protein trafficking and processing (Morel et al., 2013).

The mediation analysis of our study suggests potential roles of miR-423-5p and miR-92a-3p in AD and cognitive function. Specifically, miR-423-5p had both direct and indirect effects on AD through global cortical amyloid deposition and hippocampal volume, while miR-92a-3p did not demonstrate any significant indirect effects. These findings underscore the potential role of miR-423-5p in modulating brain Aβ deposition and neurodegeneration, suggesting its involvement in the molecular mechanisms underlying AD. The lack of significant mediation effects for miR-92a-3p suggests that its contribution to AD might involve alternative pathways or mechanisms not addressed in our analysis, which requires further exploration to fully understand its role.

The machine learning analysis for differentiating AD patients from CN individuals demonstrated that incorporating miR-423-5p and miR-92a-3p improved the classification performance of demographic information and *APOE* ε4 carrier status for AD classification from the mean AUC value of 0.708 to that of 0.757. This enhancement underscores the potential of miR-423-5p and miR-92a-3p as blood-based molecular biomarkers for AD diagnosis. Integrating these miRNA expression data with demographic information could pave the way for personalized medicine by supporting the development of strategies that account for individual differences in miRNA expression. The clinical relevance of miRNAs extends beyond their role as diagnostic biomarkers, as they are involved in the regulation of molecular pathways implicated in AD pathogenesis. This mechanistic involvement has led to growing interest in miRNA-based therapeutic strategies, including the use of synthetic miRNA mimics and anti-miRNA oligonucleotides aimed at modulating multiple gene expressions and disrupting pathological pathways (Miya Shaik et al., 2018; Bhatnagar et al., 2023).

While this study provides a comprehensive analysis of circulating miRNA expression data with central A/T/N biomarkers and cognitive function within a well-characterized cohort, several limitations need to be addressed in future studies. The modest sample size and lack of external datasets may restrict generalizability, underscoring the need for replication in future large-scale studies. This limitation also affects the clinical applicability of our miRNA-based diagnostic model, which demonstrated the modest classification performance. Moreover, information on comorbid conditions and medication use was not available in the ADNI dataset, limiting our ability to adjust for these potential confounders. Future longitudinal studies incorporating repeated miRNA measurements will be essential to elucidate the temporal dynamics underlying the observed associations and to establish causality through mediation. Finally, functional studies are crucial to unravel the specific roles of miR-423-5p and miR-92a-3p in AD pathogenesis and to validate their potential as therapeutic targets.

## 5 Conclusion

In conclusion, this study shows associations of circulating miRNAs (miR-423-5p and miR-92a-3p) with central AD biomarkers and disease progression as well as the improvement in the accuracy of AD classification, shedding light on their potential as blood-based molecular biomarkers for AD. Further investigations are warranted to validate our findings and clarify the underlying mechanisms.

## Data Availability

Publicly available datasets were analyzed in this study. This data can be found at: The ADNI cohort data will be freely available at the ADNI LONI database (http://adni.loni.usc.edu).

## Footnotes

^1^ http://adni.loni.usc.edu
