# Supplementary material for: miR-423-5p and miR-92a-3p in Alzheimer’s disease: relationship with pathology and cognition
Source: Front Aging Neurosci. 2025 Jul 22;17:1637368. doi: 10.3389/fnagi.2025.1637368 (PMC12321855; doi:10.3389/fnagi.2025.1637368)
Supplement: Supplementary file 1 [file Data_Sheet_1.docx]

**Supplementary Table 1**. Rotation sums of squares loading eigenvalues

| **PC** | **Eigenvalue** |
| --- | --- |
| **PC1** | 24.424 |
| **PC2** | 7.465 |
| **PC3** | 3.513 |
| **PC4** | 2.430 |
| **PC5** | 2.093 |
| **PC6** | 1.745 |
| **PC7** | 1.474 |
| **PC8** | 1.336 |
| **PC9** | 1.224 |
| **PC10** | 1.193 |
| **PC11** | 1.106 |
| **PC12** | 1.031 |
| **PC13** | 1.023 |

Abbreviations: PC, principal component.

**Supplementary Table 2**. Component score contribution of miRNAs to each principal component

| Rotated component matrix | | | | | | | | | | | | | |
| --- | --- | --- | --- | --- | --- | --- | --- | --- | --- | --- | --- | --- | --- |
| miRNAs | Components | | | | | | | | | | | | |
|  | PC1 | PC2 | PC3 | PC4 | PC5 | PC6 | PC7 | PC8 | PC9 | PC10 | PC11 | PC12 | PC13 |
| cel-miR-39-3p | -0.141 | 0.607 | -0.152 | 0.539 | -0.170 | 0.008 | 0.001 | 0.111 | 0.069 | 0.032 | -0.081 | 0.108 | 0.050 |
| hsa-let-7b-5p | 0.153 | 0.270 | 0.122 | 0.598 | 0.174 | 0.020 | 0.041 | 0.306 | -0.116 | -0.019 | -0.015 | -0.140 | -0.085 |
| hsa-let-7g-5p | 0.204 | 0.487 | -0.075 | 0.102 | 0.029 | 0.399 | -0.033 | 0.189 | -0.140 | 0.121 | 0.336 | -0.242 | 0.403 |
| hsa-let-7i-5p | 0.813 | 0.050 | -0.022 | 0.125 | 0.085 | 0.155 | -0.105 | 0.082 | -0.217 | -0.048 | -0.010 | -0.050 | -0.025 |
| hsa-miR-100-5p | 0.326 | 0.426 | 0.113 | 0.492 | 0.341 | -0.246 | -0.034 | 0.147 | -0.092 | -0.032 | 0.052 | 0.052 | 0.014 |
| hsa-miR-101-3p | -0.367 | 0.585 | -0.079 | 0.284 | -0.148 | 0.051 | -0.067 | -0.200 | 0.383 | 0.059 | -0.047 | -0.124 | 0.061 |
| hsa-miR-125b-5p | 0.352 | 0.676 | 0.007 | 0.090 | 0.074 | -0.046 | 0.166 | 0.075 | -0.063 | -0.061 | -0.130 | -0.138 | 0.094 |
| hsa-miR-142-3p | 0.766 | 0.300 | 0.183 | 0.055 | -0.174 | 0.281 | 0.022 | 0.030 | 0.132 | 0.063 | 0.010 | 0.011 | -0.063 |
| hsa-miR-143-3p | 0.560 | 0.048 | 0.640 | -0.012 | -0.078 | 0.102 | 0.068 | 0.037 | -0.154 | -0.016 | -0.060 | 0.044 | 0.015 |
| hsa-miR-145-5p | 0.644 | -0.007 | 0.528 | 0.102 | -0.128 | 0.015 | -0.094 | 0.174 | -0.265 | 0.071 | -0.034 | -0.031 | -0.048 |
| hsa-miR-146a-5p | 0.571 | 0.452 | 0.110 | 0.165 | -0.033 | 0.421 | -0.047 | 0.010 | -0.070 | 0.105 | 0.000 | 0.008 | 0.033 |
| hsa-miR-150-5p | -0.567 | 0.101 | -0.188 | 0.203 | -0.027 | 0.046 | -0.010 | 0.061 | 0.526 | 0.132 | 0.004 | -0.001 | -0.029 |
| hsa-miR-153-3p | 0.684 | 0.142 | 0.444 | 0.031 | 0.020 | 0.040 | 0.277 | 0.085 | -0.072 | 0.054 | -0.083 | -0.026 | -0.118 |
| hsa-miR-15a-5p | 0.254 | 0.304 | 0.320 | 0.646 | 0.076 | 0.303 | 0.068 | -0.109 | -0.028 | -0.120 | 0.058 | 0.075 | -0.111 |
| hsa-miR-16-5p | 0.108 | 0.627 | 0.073 | 0.063 | 0.249 | 0.492 | 0.076 | 0.033 | 0.175 | 0.087 | -0.059 | 0.128 | -0.154 |
| hsa-miR-181b-5p | 0.299 | 0.187 | 0.068 | 0.142 | 0.065 | 0.054 | 0.024 | 0.702 | -0.029 | 0.126 | -0.046 | -0.149 | -0.083 |
| hsa-miR-181c-5p | 0.437 | 0.630 | -0.127 | 0.117 | -0.077 | 0.231 | 0.033 | 0.120 | -0.022 | -0.010 | 0.171 | -0.115 | 0.058 |
| hsa-miR-185-5p | 0.521 | 0.464 | 0.557 | 0.121 | -0.004 | 0.183 | 0.135 | -0.058 | 0.047 | -0.047 | -0.021 | 0.075 | 0.163 |
| hsa-miR-186-5p | 0.600 | 0.131 | 0.523 | 0.321 | -0.075 | 0.235 | 0.111 | 0.107 | 0.077 | -0.003 | -0.134 | 0.074 | 0.050 |
| hsa-miR-190a-5p | 0.150 | 0.301 | 0.057 | 0.417 | 0.218 | 0.138 | 0.343 | 0.255 | 0.243 | -0.025 | -0.031 | 0.148 | 0.210 |
| hsa-miR-192-5p | 0.173 | 0.027 | 0.863 | 0.061 | 0.109 | 0.005 | 0.013 | 0.015 | -0.024 | 0.184 | 0.130 | -0.058 | -0.120 |
| hsa-miR-193a-5p | -0.001 | 0.370 | 0.133 | -0.082 | -0.142 | 0.255 | 0.282 | 0.383 | 0.084 | -0.228 | 0.216 | 0.181 | 0.058 |
| hsa-miR-194-5p | 0.082 | 0.542 | 0.351 | 0.024 | -0.017 | 0.111 | 0.239 | -0.199 | 0.238 | 0.256 | 0.103 | 0.099 | 0.216 |
| hsa-miR-195-5p | 0.273 | 0.073 | 0.886 | 0.102 | 0.013 | 0.099 | 0.105 | 0.062 | 0.119 | 0.010 | -0.129 | 0.034 | 0.031 |
| hsa-miR-199a-5p | 0.835 | 0.116 | 0.349 | 0.042 | -0.041 | 0.005 | 0.032 | 0.074 | 0.028 | 0.014 | -0.006 | -0.084 | 0.012 |
| hsa-miR-19a-3p | 0.645 | -0.019 | 0.220 | 0.019 | 0.561 | 0.025 | -0.081 | 0.118 | 0.121 | -0.088 | 0.132 | 0.038 | 0.118 |
| hsa-miR-19b-3p | 0.665 | -0.082 | 0.401 | 0.007 | 0.396 | -0.046 | -0.070 | 0.146 | 0.114 | -0.104 | 0.071 | 0.040 | 0.219 |
| hsa-miR-200a-3p | 0.662 | 0.338 | 0.252 | 0.003 | 0.183 | 0.049 | 0.195 | 0.136 | -0.161 | -0.104 | 0.002 | -0.147 | -0.089 |
| hsa-miR-204-5p | 0.384 | 0.053 | 0.244 | 0.101 | -0.058 | 0.101 | -0.106 | 0.150 | 0.031 | 0.036 | 0.271 | -0.084 | -0.592 |
| hsa-miR-210-3p | 0.464 | 0.333 | 0.449 | -0.021 | -0.040 | 0.155 | 0.009 | -0.053 | 0.054 | -0.076 | -0.070 | 0.078 | 0.341 |
| hsa-miR-2110 | 0.497 | 0.131 | 0.656 | 0.007 | -0.112 | 0.064 | 0.257 | -0.077 | -0.154 | -0.095 | -0.013 | -0.030 | 0.064 |
| hsa-miR-214-3p | -0.045 | 0.093 | 0.217 | 0.074 | -0.011 | 0.029 | 0.835 | 0.019 | 0.000 | 0.054 | 0.124 | -0.032 | 0.015 |
| hsa-miR-21-5p | 0.639 | 0.403 | 0.298 | 0.058 | 0.004 | 0.110 | 0.408 | -0.101 | -0.015 | 0.060 | 0.007 | 0.023 | 0.100 |
| hsa-miR-223-3p | 0.238 | 0.458 | 0.097 | -0.176 | -0.062 | 0.633 | 0.127 | 0.076 | 0.242 | 0.042 | -0.113 | -0.080 | 0.070 |
| hsa-miR-22-3p | 0.217 | 0.080 | 0.318 | 0.170 | 0.054 | 0.797 | 0.000 | 0.024 | -0.138 | 0.052 | 0.053 | 0.058 | -0.067 |
| hsa-miR-23a-3p | 0.797 | 0.053 | 0.088 | 0.094 | 0.235 | 0.086 | -0.233 | 0.077 | -0.140 | -0.081 | 0.095 | -0.066 | 0.031 |
| hsa-miR-24-3p | 0.270 | 0.701 | 0.079 | 0.226 | 0.193 | 0.289 | 0.057 | 0.086 | -0.005 | 0.004 | -0.099 | 0.166 | -0.159 |
| hsa-miR-26a-5p | 0.762 | 0.157 | 0.356 | 0.047 | -0.092 | 0.362 | 0.186 | 0.049 | 0.081 | 0.010 | -0.018 | 0.078 | -0.037 |
| hsa-miR-26b-5p | 0.152 | 0.363 | 0.338 | 0.608 | -0.144 | 0.063 | 0.052 | -0.104 | 0.024 | -0.021 | 0.054 | 0.026 | 0.018 |
| hsa-miR-27b-3p | 0.858 | 0.044 | 0.096 | 0.076 | 0.071 | -0.030 | -0.019 | 0.138 | 0.014 | -0.026 | 0.031 | 0.010 | 0.077 |
| hsa-miR-29a-3p | 0.640 | 0.332 | 0.273 | 0.023 | 0.437 | 0.083 | 0.022 | 0.172 | -0.007 | 0.003 | 0.026 | -0.107 | -0.141 |
| hsa-miR-29b-3p | 0.444 | 0.343 | 0.627 | 0.084 | 0.032 | 0.291 | 0.038 | 0.036 | -0.036 | 0.066 | -0.082 | 0.022 | -0.074 |
| hsa-miR-29c-3p | 0.340 | -0.038 | 0.810 | 0.146 | 0.055 | 0.052 | 0.084 | 0.004 | -0.163 | -0.041 | 0.019 | -0.065 | -0.044 |
| hsa-miR-30a-3p | -0.484 | 0.109 | -0.326 | 0.059 | 0.032 | -0.278 | -0.111 | 0.303 | 0.004 | 0.018 | -0.157 | 0.211 | 0.104 |
| hsa-miR-30b-5p | 0.785 | -0.016 | 0.450 | -0.001 | -0.075 | 0.143 | 0.142 | 0.000 | -0.153 | -0.021 | -0.041 | -0.078 | -0.092 |
| hsa-miR-30d-5p | -0.428 | 0.448 | -0.364 | 0.233 | 0.472 | 0.075 | 0.024 | -0.113 | 0.131 | 0.042 | 0.166 | 0.061 | -0.015 |
| hsa-miR-323a-3p | 0.721 | 0.172 | 0.088 | 0.082 | -0.135 | 0.010 | -0.091 | -0.062 | -0.095 | -0.009 | 0.017 | 0.085 | -0.125 |
| hsa-miR-338--3p | 0.261 | 0.631 | 0.186 | 0.056 | -0.162 | 0.166 | 0.044 | 0.166 | 0.142 | -0.282 | -0.153 | 0.112 | -0.071 |
| hsa-miR-342-3p | 0.597 | 0.591 | 0.241 | -0.054 | 0.065 | -0.043 | -0.024 | 0.082 | 0.181 | 0.065 | 0.022 | -0.117 | 0.080 |
| hsa-miR-374b-5p | 0.860 | 0.238 | 0.129 | 0.043 | 0.163 | 0.002 | 0.126 | 0.135 | 0.066 | -0.013 | 0.054 | -0.035 | 0.022 |
| hsa-miR-376a-3p | 0.732 | 0.169 | 0.236 | 0.186 | 0.068 | 0.100 | -0.079 | -0.025 | 0.023 | -0.060 | 0.091 | -0.053 | -0.079 |
| hsa-miR-378a-3p | 0.401 | 0.166 | 0.420 | 0.077 | 0.191 | 0.139 | -0.090 | 0.368 | -0.016 | -0.302 | -0.060 | 0.052 | -0.009 |
| hsa-miR-423-5p | 0.124 | 0.808 | 0.044 | 0.084 | 0.095 | 0.155 | 0.038 | 0.088 | 0.075 | -0.063 | -0.051 | 0.078 | -0.002 |
| hsa-miR-424-5p | 0.021 | 0.358 | 0.477 | 0.076 | 0.029 | 0.097 | -0.115 | 0.233 | 0.402 | -0.052 | 0.110 | -0.071 | -0.193 |
| hsa-miR-425-5p | 0.144 | 0.705 | 0.165 | 0.116 | -0.246 | -0.218 | -0.144 | 0.028 | -0.110 | 0.037 | 0.097 | -0.136 | 0.322 |
| hsa-miR-484 | 0.653 | 0.363 | 0.432 | 0.203 | -0.070 | 0.282 | 0.144 | -0.125 | -0.018 | -0.016 | -0.040 | -0.009 | -0.019 |
| hsa-miR-499a-3p | 0.068 | -0.060 | -0.056 | 0.012 | 0.056 | 0.003 | 0.117 | -0.035 | 0.009 | -0.081 | 0.812 | 0.120 | -0.079 |
| hsa-miR-502-3p | 0.316 | 0.279 | 0.617 | 0.130 | 0.379 | 0.122 | 0.132 | 0.070 | -0.086 | -0.104 | -0.031 | -0.081 | -0.052 |
| hsa-miR-532-5p | 0.261 | 0.221 | 0.367 | 0.241 | -0.019 | 0.420 | -0.024 | 0.125 | 0.195 | -0.011 | 0.132 | 0.208 | 0.105 |
| hsa-miR-647 | -0.116 | 0.084 | -0.049 | 0.025 | 0.011 | 0.051 | -0.010 | -0.071 | 0.007 | 0.097 | 0.113 | 0.835 | 0.021 |
| hsa-miR-660-5p | 0.015 | 0.745 | 0.219 | 0.154 | 0.128 | 0.054 | 0.068 | 0.060 | -0.205 | 0.065 | 0.050 | 0.043 | -0.128 |
| hsa-miR-885-5p | -0.067 | 0.033 | 0.061 | -0.060 | -0.033 | 0.087 | 0.043 | 0.060 | 0.068 | 0.858 | -0.087 | 0.103 | -0.014 |
| hsa-miR-92a-3p | 0.141 | 0.841 | 0.143 | 0.208 | 0.052 | 0.016 | 0.018 | 0.026 | -0.128 | 0.033 | 0.035 | 0.062 | -0.043 |
| hsa-miR-92b-3p | 0.203 | 0.341 | 0.273 | 0.339 | -0.147 | 0.014 | -0.085 | 0.110 | -0.625 | -0.088 | 0.016 | -0.079 | 0.011 |

Abbreviations: miR, microRNA; PC, principal component.

**Supplementary Table 3**. Association analysis results of PCs with diagnostic groups (CN vs. AD)

|  | **AD diagnosis** | |
| --- | --- | --- |
| **PC** | **OR (95% CI)** | **FDR-corrected *P* value** |
| **PC1** | 1.21 (0.87 – 1.70) | 0.464 |
| **PC2** | 0.59 (0.41 – 0.75) | **0.038** |
| **PC3** | 1.05 (0.75 – 1.47) | 0.902 |
| **PC7** | 1.22 (0.88 – 1.74) | 0.464 |
| **PC10** | 0.72 (0.49 – 1.01) | 0.243 |
| **PC11** | 0.99 (0.71 – 1.38) | 0.958 |
| **PC12** | 0.87 (0.62 – 1.21) | 0.564 |

Abbreviations: AD, Alzheimer’s disease; CI, confidence interval; CN, cognitively normal; FDR, false discovery rate; OR, odds ratio; PC, principal component.

**Supplementary Table 4**. Association analysis results of PC2 with amyloid, tau, and neurodegeneration (A/T/N) biomarkers

| **Biomarkers** | | **β ^a^** | **Standard Error** | ***P* value** |
| --- | --- | --- | --- | --- |
| **A** | **Amyloid PET global SUVR** | -0.012 | 0.007 | 0.083 |
| **A** | **CSF Aβ42** | 0.027 | 0.020 | 0.189 |
| **T** | **CSF p-tau** | -0.035 | 0.016 | 0.033 |
| **N** | **Hippocampal volume** | 90.105 | 39.647 | 0.025 |

Due to missing values, the number of participants excluded from the analysis was 3 for amyloid PET global SUVR, 1 for CSF Aβ42 levels, 2 for CSF p-tau levels, and 2 for hippocampal volume.

^a^ Linear regression coefficients of association between PC2 and A/T/N biomarkers

Abbreviations: Aβ, amyloid-β; CSF, cerebrospinal fluid; p-tau, phosphorylated tau; PC, principal component; PET, positron emission tomography; SUVR, standardized uptake value ratio.

**Supplementary Table 5**. Association analysis results of PC2 with cognition at baseline and longitudinal change of cognition

|  | **Baseline** | | | **Longitudinal** | | |
| --- | --- | --- | --- | --- | --- | --- |
|  | **β ^a^** | **Standard Error** | ***P* value** | **β ^b^** | **Standard Error** | ***P* value** |
| **Composite score for memory** | 0.222 | 0.074 | 0.003 | 0.234 | 0.086 | 0.008 |
| **Composite score for executive function** | 0.135 | 0.064 | 0.038 | 0.122 | 0.072 | 0.090 |

^a^ Linear regression coefficients of association between PC2 and cognitive traits

^b^ Coefficients of association between PC2 and cognitive traits from linear mixed effects models

Abbreviations: PC, principal component.

**Supplementary Table 6**. Association analysis results of miR-423-5p and miR-92a-3p with cognition at baseline and longitudinal change of cognition

|  | **Baseline** | | | | | | **Longitudinal** | | | | | |
| --- | --- | --- | --- | --- | --- | --- | --- | --- | --- | --- | --- | --- |
|  | **miR-423-5p** | | | **miR-92a-3p** | | | **miR-423-5p** | | | **miR-92a-3p** | | |
|  | **β ^a^** | **Standard Error** | ***P* value** | **β ^a^** | **Standard Error** | ***P* value** | **β ^b^** | **Standard Error** | ***P* value** | **β ^b^** | **Standard Error** | ***P* value** |
| **Composite score for memory** | 20.164 | 6.098 | 0.001 | 13.652 | 4.941 | 0.006 | 21.573 | 7.133 | 0.003 | 15.684 | 5.775 | 0.007 |
| **Composite score for executive function** | 13.348 | 5.317 | 0.013 | 9.624 | 4.280 | 0.026 | 12.483 | 5.927 | 0.037 | 9.276 | 4.799 | 0.055 |

^a^ Linear regression coefficients of association between miRNAs and cognitive traits

^b^ Coefficients of association between miRNAs and cognitive traits from linear mixed effects models

Abbreviations: miR, microRNA.

**Supplementary Table 7**. Results of the mediation analysis of miR-423-5p and miR-92a-3p on AD diagnosis and composite scores for memory

|  | | | **Mediation effect** | | **Direct effect** | | **Total effect** | |
| --- | --- | --- | --- | --- | --- | --- | --- | --- |
| **Independent**  **variable** | **Dependent**  **variable** | **Mediator** | **Estimate**  **(95% CI)** | ***P*** | **Estimate**  **(95% CI)** | ***P*** | **Estimate**  **(95% CI)** | ***P*** |
| **miR-423-5p** | **AD** | **Amyloid PET global SUVR** | -2.61 × 10^-10^  (-2.18 × 10^-2^ – 0.00) | 0.02 ^a^ | -2.61 × 10^-10^  (-1.92 × 10^-3^ – 0.00) | 0.04 ^a^ | -5.22 × 10^-10^  (-8.47 × 10^-3^ – 0.00) | 0.02 ^a^ |
|  |  | **Hippocampal volume** | -9.86 × 10^-16^  (-0.31 – 0.00) | 0.02 ^b^ | -9.86 × 10^-16^  (-5.23 × 10^-3^ – 0.36) | 0.16 ^b^ | -1.97 × 10^-15^  (-1.40 × 10^-2^ – 0.00) | 0.02 ^b^ |
|  | **Composite scores for memory** | **Amyloid PET global SUVR** | 4.44  (0.25 – 9.26) | 0.04 ^c^ | 10.29  (2.44 – 18.67) | 0.02 ^c^ | 14.74  (3.90 – 23.55) | < 0.001 ^c^ |
|  |  | **Hippocampal volume** | 7.80  (1.47 – 13.26) | 0.04 ^d^ | 8.52  (2.52 – 15.04) | 0.02 ^d^ | 16.31  (7.79 – 26.16) | < 0.001 ^d^ |
| **miR-92a-3p** | **AD** | **Hippocampal volume** | -0.09  (-0.37 – 0.51) | 0.72 ^b^ | -0.16  (-0.55 – 0.42) | 0.34 ^b^ | -0.26  (-0.32 – 0.26) | 0.32 ^b^ |
|  | **Composite scores for memory** | **Hippocampal volume** | 0.57  (-1.64 – 3.60) | 0.64 ^d^ | 2.06  (0.27 – 1.28) | 0.04 ^d^ | 2.63  (0.05 – 7.25) | 0.04 ^d^ |

^a^ After adjusting for age, sex, and *APOE* ε4 carrier status

^b^ After adjusting for age, sex, and intracranial volume

^c^ After adjusting for age, sex, years of education, and *APOE* ε4 carrier status

^d^ After adjusting for age, sex, years of education, and intracranial volume

Abbreviations: AD, Alzheimer’s disease; *APOE*, apolipoprotein E; CI, confidence interval; miR, microRNA; PET, positron emission tomography; SUVR, standard uptake value ratio.
